# Side-to-side magnet anastomosis system duodeno-ileostomy with sleeve gastrectomy: early multi-center results

**DOI:** 10.1007/s00464-023-10134-6

**Published:** 2023-05-22

**Authors:** Michel Gagner, Guy-Bernard Cadiere, Andres Sanchez-Pernaute, David Abuladze, Todd Krinke, J. N. Buchwald, Nathalie Van Sante, Marc Van Gossum, Jana Dziakova, Levan Koiava, Maja Odovic, Mathilde Poras, Lamees Almutlaq, Antonio J. Torres

**Affiliations:** 1 Westmount Square Surgical Center, Westmount, QC Canada; 2 CHU St-Pierre, Brussels, Belgium; 3 Hospital Clinico San Carlos, Madrid, Spain; 4 Innova Medical Center, Tbilisi, Republic of Georgia; 5 GT Metabolic Solutions, San Jose, CA USA; 6 Medwrite Medical Communications, Maiden Rock, WI USA; 7 NVS Consulting, Brussels, Belgium; 8 Westmount Square Surgical Center, 1 Westmount Square, Suite 801, Westmount, QC H3Z2P9 Canada

**Keywords:** Metabolic/bariatric surgery, Magnetic compression anastomosis, Magnet system, Duodeno-ileostomy, Sleeve gastrectomy, Obesity, Type 2 diabetes

## Abstract

**Introduction:**

Gastrointestinal anastomoses with classical sutures and/or metal staples have resulted in significant bleeding and leak rates. This multi-site study evaluated the feasibility, safety, and preliminary effectiveness of a novel linear magnetic compression anastomosis device, the Magnet System (MS), to form a side-to-side duodeno-ileostomy (DI) diversion for weight loss and type 2 diabetes (T2D) resolution.

**Methods:**

In patients with class II and III obesity (body mass index [BMI, kg/m^2^] ≥ 35.0– ≤ 50.0 with/without T2D [HbA1C > 6.5%]), two linear MS magnets were delivered endoscopically to the duodenum and ileum with laparoscopic assistance and aligned, initiating DI; sleeve gastrectomy (SG) was added. There were no bowel incisions or retained sutures/staples. Fused magnets were expelled naturally. Adverse events (AEs) were graded by Clavien-Dindo Classification (CDC).

**Results:**

Between November 22, 2021 and July 18, 2022, 24 patients (83.3% female, mean ± SEM weight 121.9 ± 3.3 kg, BMI 44.4 ± 0.8) in three centers underwent magnetic DI. Magnets were expelled at a median 48.5 days. Respective mean BMI, total weight loss, and excess weight loss at 6 months (n = 24): 32.0 ± 0.8, 28.1 ± 1.0%, and 66.2 ± 3.4%; at 12 months (n = 5), 29.3 ± 1.5, 34.0 ± 1.4%, and 80.2 ± 6.6%. Group mean respective mean HbA1_C_ and glucose levels dropped to 1.1 ± 0.4% and 24.8 ± 6.6 mg/dL (6 months); 2.0 ± 1.1% and 53.8 ± 6.3 mg/dL (12 months). There were 0 device-related AEs, 3 procedure-related serious AEs. No anastomotic bleeding, leakage, stricture, or mortality.

**Conclusion:**

In a multi-center study, side-to-side Magnet System duodeno-ileostomy with SG in adults with class III obesity appeared feasible, safe, and effective for weight loss and T2D resolution in the short term.

Metabolic/bariatric surgery (MBS) is substantially more effective and durable in achieving weight loss and type 2 diabetes control than conventional lifestyle change and medication [1–3]. MBS is also highly cost effective in managing patients with T2D with/without obesity, saving insurers > $76.5 million in T2D medication costs in the first post-MBS year in one state alone [4]. MBS is safe, with approximately the same short-term morbidity of common procedures such as cholecystectomy and appendectomy [5, 6]. Since the introduction of laparoscopic technique in Roux-en-Y gastric bypass (RYGB) by Wittgrove and Clark (1993) [7, 8], minimally invasive surgery (MIS) has continued to reduce operative and long-term MBS risk [9, 10] and encompass novel modes of access (e.g., endoscopic, natural orifice transluminal endoscopic surgery [NOTES], robotic), technique simplification (e.g., single-anastomosis, staging/dividing complex operations), and innovative technology (e.g., intragastric balloon, gastric electrical stimulation, duodenojejunal bypass liner) [11, 12].

Forming and protecting the anastomosis must be an essential minimally invasive MBS focus. Compression anastomosis (CA) creation, performed as early as 1826 with a sutureless metal bowel ring by Denan [13], has been applied effectively in twenty-first century duodeno-ileal (DI) and duodeno-colic (DC) preclinical procedures by Gagner et al. [14, 15], and clinically, in the biofragmentable anastomotic ring (BAR) by Hardy et al., and the nickel-titanium ring and clip (NiTi CAR/CAC) reported by Nudelman et al. [16–18]. Although CA devices have shown efficacy equivalent to conventional sutures and staples [19, 20], they require fixation in the bowel with sutures, staples, clips, or glue which may stimulate an inflammatory response.

Alternatively, *magnetic* CA (MCA) eliminates sharp bowel division followed by sewing/stapling to form the anastomosis, relying instead on magnetic compression with no anatomical fixation. MCA is initiated intraoperatively by alignment of magnets across two GI bowel segments. The gradually fusing magnets necrose and slough the interposed tissue over several weeks while healing occurs at the magnets’ circumference. Magnets are expelled naturally leaving no foreign materials in the body [20, 21].

Few MCA outcomes in MBS have been reported. We performed a preclinical feasibility study of MCA [22] with favorable outcomes that supported conduct of a first-in-human (FIH) trial of the novel Magnet Anastomosis System (MS) in a side-to-side duodeno-ileal (DI) diversion with an added sleeve gastrectomy (SG) [23]. Magnetic DI diversion performed side-to-side coupled with SG may provide the strong weight loss and associated medical condition (AMC) resolution of duodenal switch (DS) without its hypoabsorption, and the added MIS advantage of a single-anastomosis DI with SG (SADI-S). In the current study, the scope of the FIH safety investigation was expanded to examine the feasibility, ongoing safety, and early efficacy of side-to-side MS DI + SG in a small cohort in multiple centers.

## Patients and methods

### Study design and protocol

A prospective observational open-label evaluation (Clinicaltrials.gov NCT#05,322,122) of the investigational Magnet Anastomosis System (MS; GT Metabolic Solutions, San Jose, CA) used in side-to-side magnetic DI for patients with class III obesity (body mass index [BMI, kg/m^2^] ≥ 40.0 to ≤ 50.0) was conducted in two stages. Stage 1 (n = 5) evaluated FIH feasibility and safety outcomes at a single site (Republic of Georgia); stage 2 (the current study) evaluated feasibility, ongoing safety, and efficacy (n = 24) in the first site plus two additional centers (Georgia, n = 5; Belgium, n = 9; Spain, n = 10) at interim time points through one year.

### Ethics

The protocol incorporated guidelines for use of investigational devices. The Ethics Committees and IRBs of the three centers approved the protocol and monitored the progress of the study. Adverse events (AEs) and severe AEs (SAEs) were analyzed during the conduct of the study by an independent data and safety monitoring board (DSMB). The Helsinki Declaration and ISO14155 regulations and 21 CFR Good Clinical Practices ensured patients’ protection and wellbeing throughout the study.

### Inclusion and exclusion

The surgeon and surgical team at the participating centers introduced potential patients to the study aims, procedure, and expected outcomes. Based on a reasonable understanding of the background, each participating patient provided written informed consent for study participation.

Inclusion criteria required patients to be 18–65 years old with a BMI of ≥ 30.0 to ≤ 50.0 kg/m^2^ and either: type 2 diabetes (T2D: HbA1_C_ ≥ 6.5% on diabetes medication) with no prior MBS; *or* prior sleeve gastrectomy (SG) ≥ 12 months previously with T2D with/without weight regain; *or* have a BMI ≥ 40 kg/m^2^ and be considering undergoing laparoscopic SADI-S where DI was performed side to side rather than end to side. Patients agreed not to undergo additional MBS or reconstructive surgery for 1 year; females agreed not to become pregnant and to use contraception for 1 year. Prescription or over-the-counter weight-loss medication and non-steroidal anti-inflammatory drugs were prohibited for 14 days before the procedure and during the study.

Exclusions to participation included: type 1 diabetes; uncontrolled T2D, dyslipidemia, hypertension, and sleep apnea; injectable insulin use; prior non-MBS intestinal, colonic, or duodenal surgery; prior trauma, implant of prostheses, disease, scarring, abnormal anatomy, or genetic preconditions that prevented or contraindicated the study procedure; refractory gastroesophageal reflux disease (GERD), helicobacter pylori positive and/or active ulcer disease; large hiatal hernia; inflammatory bowel disease or colonic diverticulitis; an anomaly or condition precluding access by gastroscopy or laparoscopy; implantable pacemaker or defibrillator; untreated or poorly controlled psychiatric illness; substance abuse history; pregnancy, breastfeeding, or unwillingness to use a proven contraception method; an AMC that presented a safety concern; an interventional procedure 30 days before/after the procedure; stroke/TIA within 6 months of study consent; chronic anticoagulation therapy (except aspirin); active infection requiring antibiotics; inability to comply with the follow-up schedule; participation in a separate clinical investigation; known allergies to device components or contrast media; limited life expectancy due to terminal disease; positive Covid-19 test before the procedure; and any condition that might prevent 360-day follow-up.

### MS DI procedure

**Fig. 1:**
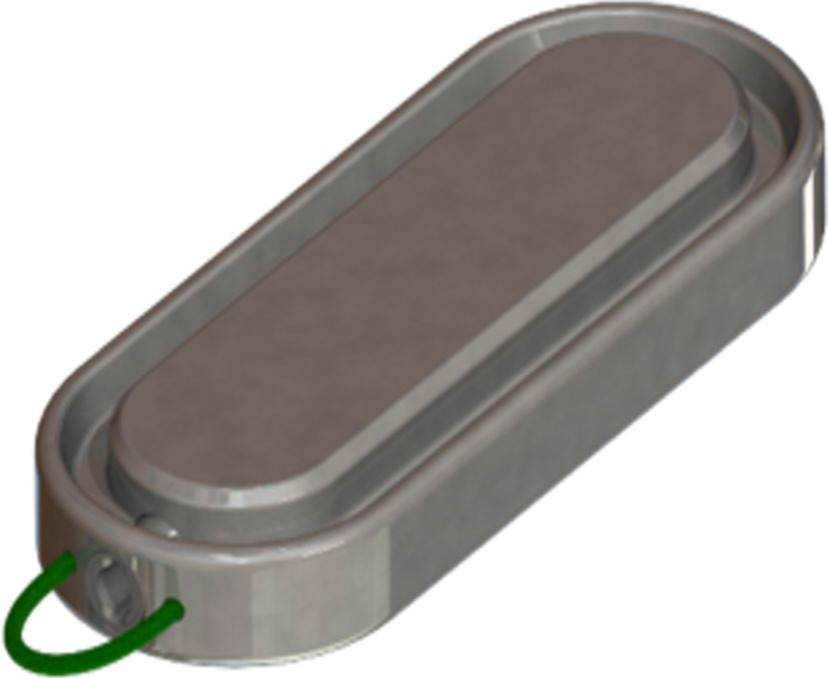
Single magnet of the Magnet Anastomosis System (MS)

**Fig. 2:**
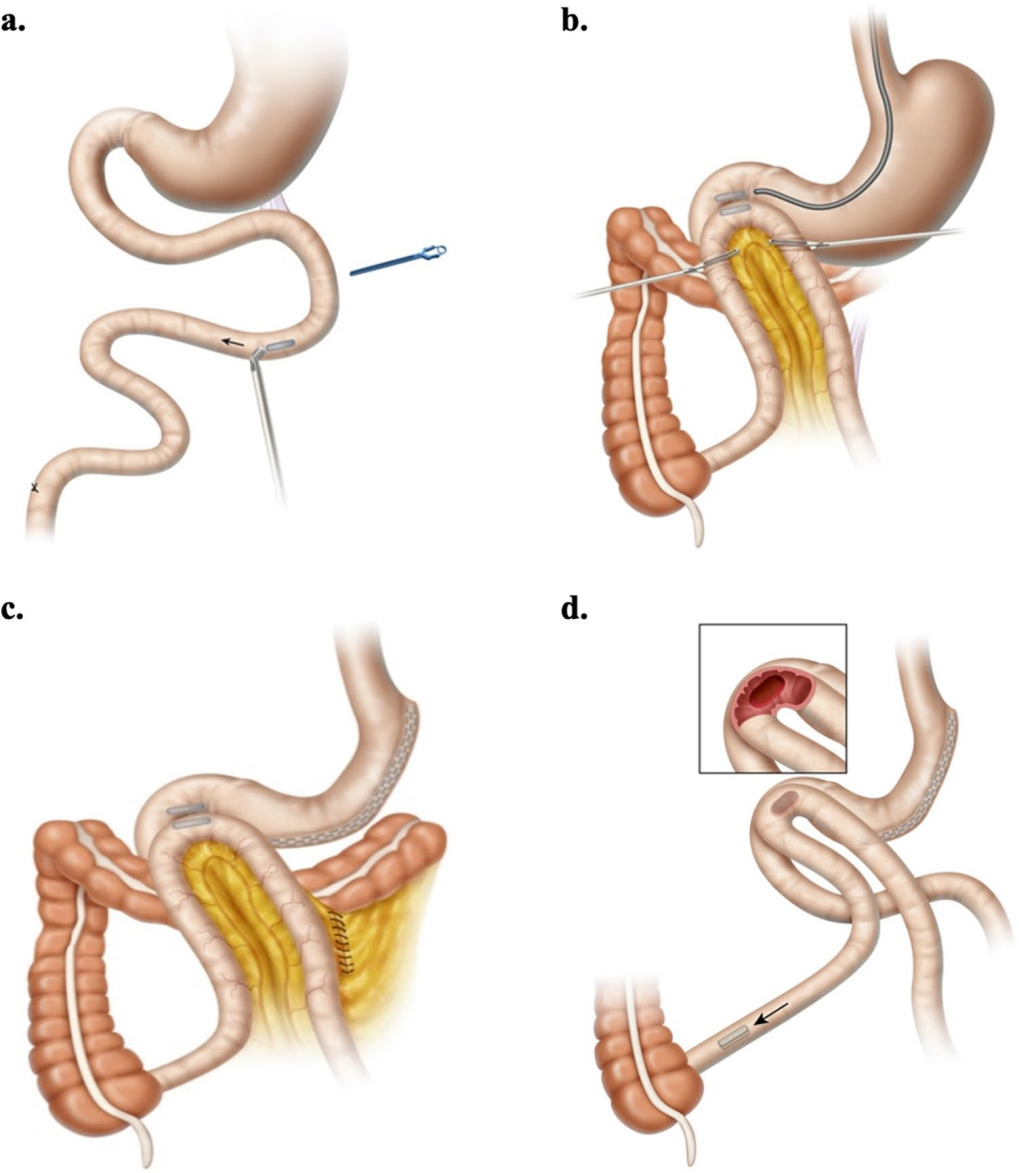
Side-to-side Magnet System (MS) duodeno-ileostomy: the distal MS magnet is endoscopically positioned at the ligament of Treitz and directed through the jejunal lumen to the ileum with laparoscopic assistance by a magnetic positioning device (**a**); the distal magnet is raised anterior and latero-lateral to the post-pyloric duodenum to align with the endoscopically delivered proximal MS magnet **(b)**; Petersen’s defect is closed and a sleeve gastrectomy is performed **(c)**. In 5–7 days magnets are fused; in several weeks, the patent DI anastomosis is formed; the magnet pair is expelled naturally, and food transits through duodenum and ileum (**d**)

Comprised of a pair of linear BC42 neodymium magnets (0.75″ length × 0.25″ width × 0.125″ thickness) with 2.3-mm-offset perimeter flange and Ti-6Al-4 V ELI grade-23 titanium casing (KJ Magnetics, Pipersville, PA) (Fig. 1), the MS was designed specifically to effect side-to-side DI diversion. The full endoscopic technique with laparoscopic assistance for side-to-side MS DI has been described [23]. In brief, after placing a marker in the ileum 250 cm from the cecum, the first (distal) MS magnet is transported orogastrically to the ligament of Treitz, then directed through the jejunal lumen by a magnetized positioning device to the marked ileal position (Fig. 2a); non-magnetic bowel forceps raise the distal ileal magnet over the transverse colon, anterior and latero-lateral to the post-pyloric duodenum, where the proximal MS magnet has been delivered endoscopically and the two magnets are aligned (Fig. 2b); Petersen’s defect is closed and an SG is performed (Fig. 2c).

In 5–7 days, magnets are fused. Within several weeks, a robust, patent anastomosis is formed and the magnet pair detaches from the DI site and is expelled naturally enabling the transit of food through the duodenal lumen and the ileal lumen (Fig. 2d).

### Post-procedure care

Patients’ hemodynamic conditions and cardiac rhythm were monitored closely following the procedure. Correct MS placement in the abdomen was confirmed on postoperative day 1 by x-ray, and by fluoroscopy using barium or gastrographin. Before discharge, patients discussed the postoperative diet with the dietitian or nutritionist. Patients attended six follow-up visits (day 30, 60, 90, 180, 270, and 360), returning as needed for consultations.

### Endpoints

Feasibility was the primary endpoint assessed by (1) correct technical positioning of the MS magnets, (2) magnet expulsion with no device-related AEs requiring surgical reintervention, and (3) patent DI anastomosis creation verified radiologically and fluoroscopically. If the MS performed appropriately in ≥ 80% of patients, the primary feasibility endpoint was considered achieved. Incidence of device- and procedure-related AEs/SAEs at 6 months and 1 year represented the primary safety endpoints. Adverse events were characterized using the Clavien-Dindo Classification (CDC) [24].

Secondary endpoints were measures of MBS efficacy including weight loss and improvement in T2D as measured by reduction or elimination of medications and improvement of metabolic markers at 6- and 12-month follow-up including the proportion of patients with > 5.0% total weight loss (TWL). TWL was calculated by: ([initial weight − follow-up weight]/[initial weight] × 100); excess weight loss (%EWL) by: ([initial weight − follow-up weight]/[initial weight – ideal body weight] × 100); and BMI loss: (initial BMI – post-intervention BMI).

### Statistical analysis

Descriptive statistics were calculated using the SPSS statistical package (version 20.0; IBM, Chicago, IL). Primary endpoints were represented by categorical variables and reported using frequencies and percentages. Summary statistics for continuous variables were reported using means and standard error of the mean (SEM), ranges and/or 95% CIs. Group mean changes in weight and metabolic parameters were assessed using the paired samples t-test; alpha was set at p < 0.05.

## Results

### Patient characteristics

Between November 22, 2021 and July 18, 2022, 24 patients (83.3% female; 75.0% Caucasian) with a mean age of 43.8 ± 1.8 years (range 28–59) underwent side-to-side MS DI + SG. The group mean baseline absolute weight was 121.9 ± 3.3 kg (97–155) with a BMI of 44.4 ± 0.8 (36.8–50.9) (Table 1). Nine patients (37.5%) were diagnosed with T2D, most on medications, at study enrollment. (Three patients, one in an included site, two with 3-month follow-up in a site that was not included in this study, did not receive an SG concurrent with the DI and are incorporated in an independent report.)

**Table 1:** Patient characteristics and perioperative outcomes

Characteristics	N = 24
Preoperative	
Age, yrs, mean ± SEM (range)	43.8 ± 1.8 (28.0–59.0)
Females, n (%)	20 (83.3)
Ethnicity	
Caucasian, n (%)	18 (75.0)
Not offered, n (%)	6 (25.0)
Height (cm), mean ± SEM (range	165.5 ± 1.6 (154.0–185.0)
Weight, kg, mean ± SEM (range)	121.9 ± 3.3 (97.0–155.0)
BMI, kg/m^2^, mean ± SEM (range)	44.4 ± 0.8 (36.8–50.9)
Waist circumference (cm), mean ± SEM (range)	128.1.3 ± 2.7 (109.0–163.0)
Ideal weight kg, mean ± SEM (range)	68.6 ± 1.4 (59.3–85.6)
Excess weight kg, mean ± SEM (range)	53.4 ± 2.4 (32.2–71.1)
Type 2 diabetes mellitus, n (%)	9 (37.5)
HbA1_C_, %, mean ± SEM (range)	6.2 ± 0.3 (5.0–10.0)
Glucose, mg/dL, mean ± SEM (range)	112.7 ± 5.8 (82.0–178.8)
Perioperative	
Operative time, mean ± SEM (range)	128 ± 0.1 (1.0–3.15)
Hospital stay, days, mean ± SEM (range)	6 ± 1.7 (2–40)*
Expulsion of magnets, days, median (range)	48.5 (14.0–92.0)
Expulsion mean ± SEM (range)	48.2 ± 4.7 (14.0–92.0)

*BMI*: Body mass index; *HbA1*_*C*_: Glycosylated hemoglobin; *SEM*: Standard error of the mean

^*^Outliers: (severe adverse events = 24 days, pelvic collection (first episode); 40 days, pneumoperitoneum + pneuomonia

### Feasibility

**Fig. 3:**
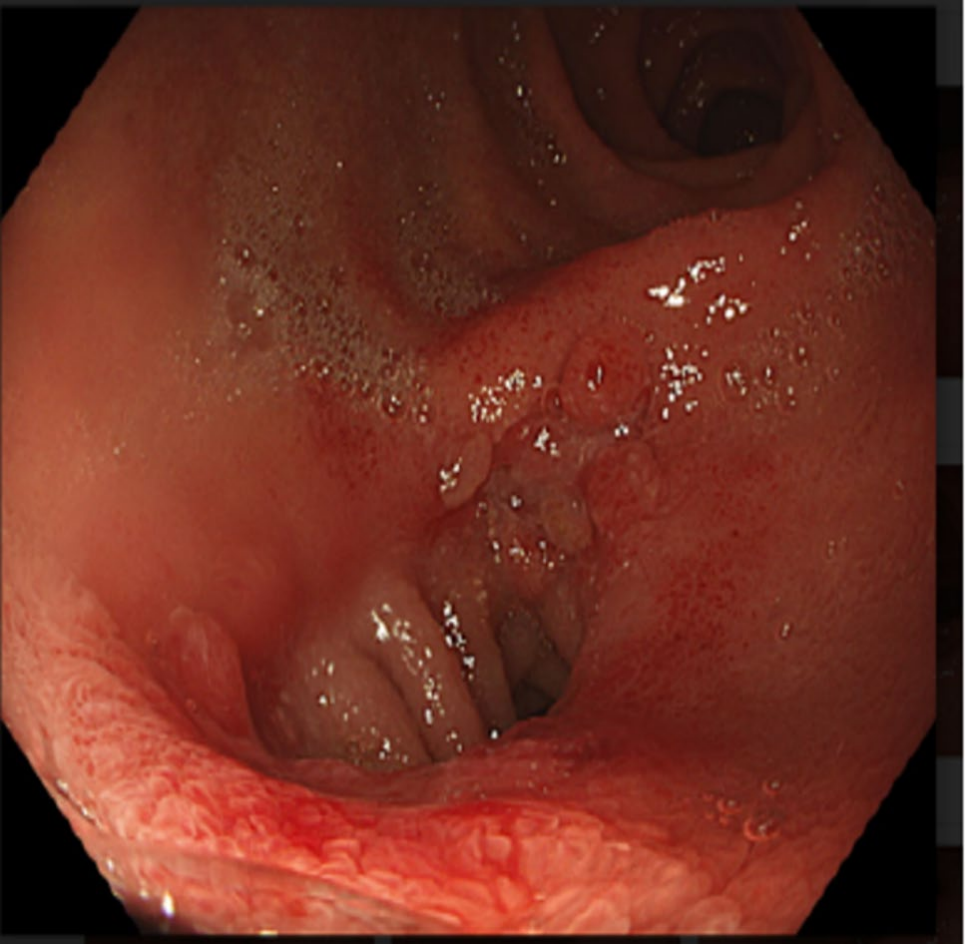
Endoscopic view (from pylorus toward first part of duodenum) of patent side-to-side duodeno-ileal anastomosis formed with the Magnet System

The MS successfully met feasibility criteria: placement of the device with alignment of magnets, creation of a patent anastomosis confirmed radiologically (Fig. 3), and passage of the paired magnets without AEs requiring re-intervention was achieved in all 24 (100.0%) patients. The time to device expulsion (subject to self-report) ranged from 14 to 92 days with a median of 48.5 days (n = 22; mean 48.2 ± 4.7 days). Two patients indicated they did not note the natural passage of the magnets; however, expulsion was subsequently confirmed through imaging.

### Secondary efficacy endpoints

#### Weight

**Fig. 4:**
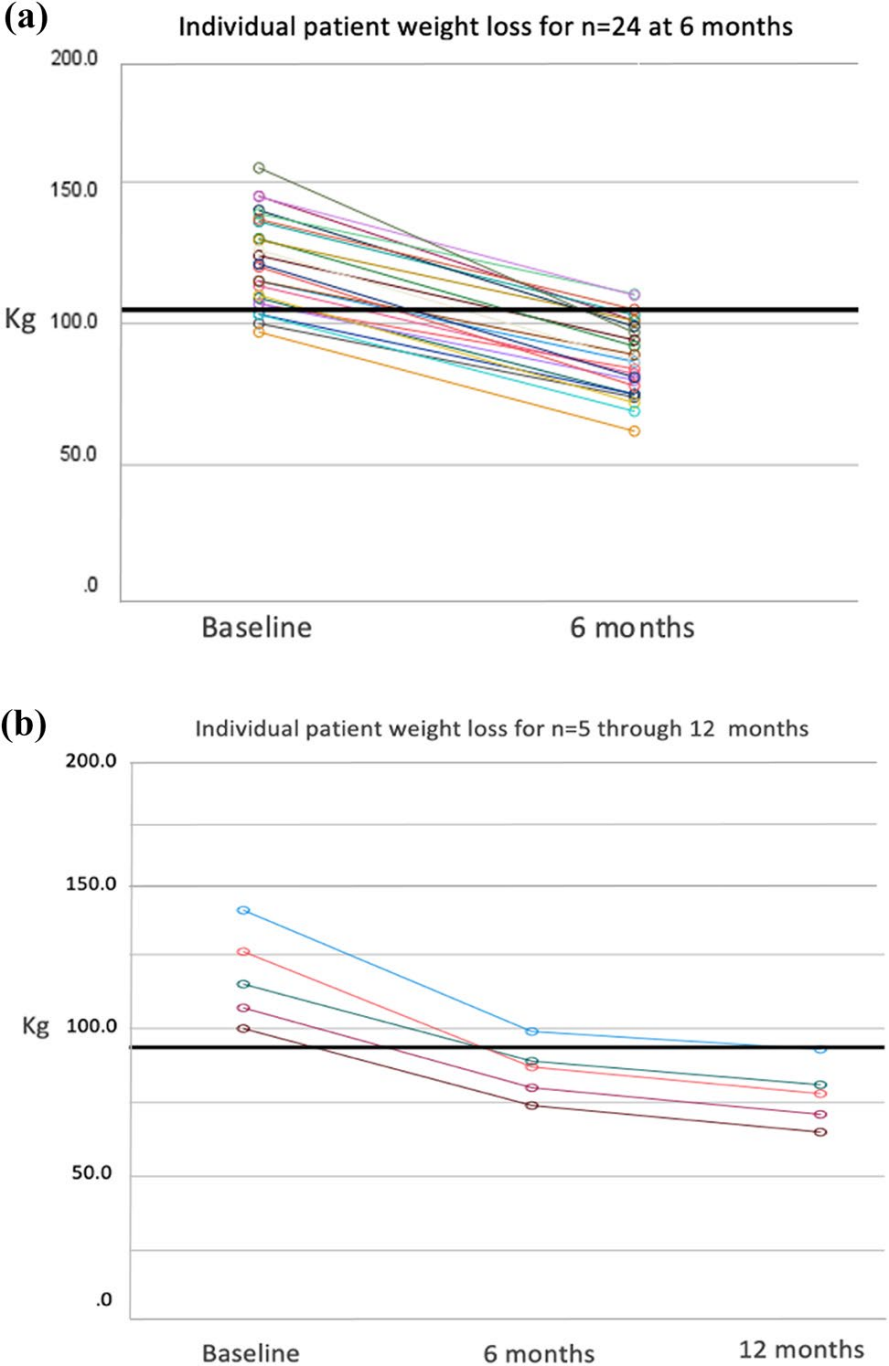
Individual patient absolute weight loss (kg) trajectories following side-to-side Magnet System duodeno-ileostomy with sleeve gastrectomy from baseline (**a**) to 6 months (n = 24) and (**b**) to 12 months (n = 5)

**Fig. 5:**
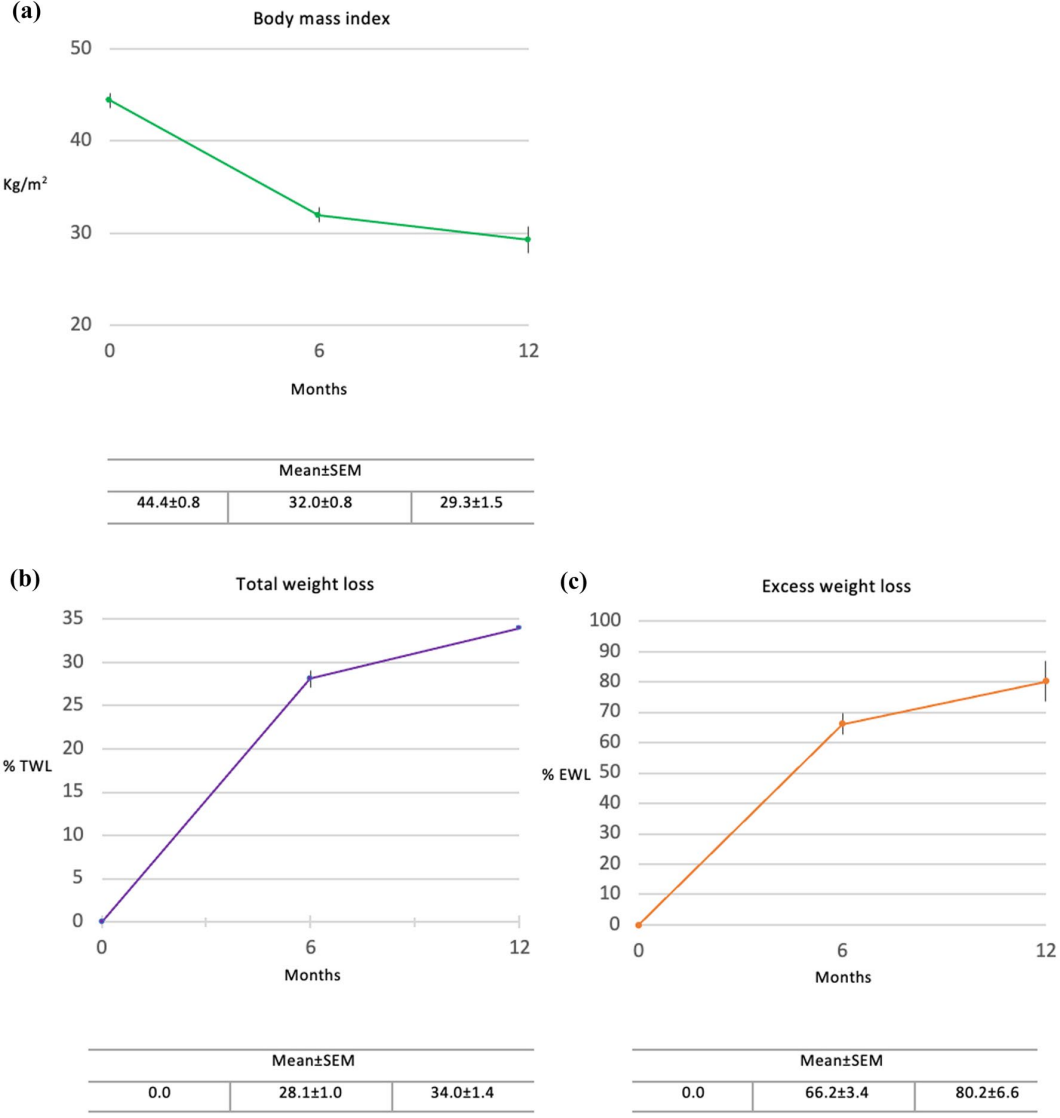
Mean changes in weight from baseline to 6 months (n = 24) and 12 months (n = 5) following side-to-side Magnet System duodeno-ileostomy with sleeve gastrectomy in **(a)** body mass index (BMI, kg/m^2^); (**b**) total weight loss (%TWL); and in (**c**) excess weight loss (%EWL)

Group mean body weight (n = 24) was reduced from 121.9 ± 3.3 kg at baseline to 87.8 ± 2.8 kg at day 180, representing an overall mean weight change of 34.2 ± 1.6 kg (p < 0.001) (Table 2). For the five patients who reached the 360-day follow-up, mean body weight fell to 77.6 ± 4.7 kg, an overall reduction of 40.0 ± 3.1 kg (p < 0.001). Figure 4a and b depict individual patient weight-loss trajectories to day 180 (n = 24) and day 360 (n = 5), respectively. As presented in Table 2 and Fig. 5a, BMI fell significantly from 44.4 ± 0.8 at baseline to 32.0 ± 0.8 (p < 0.001) at day 180 (n = 24) and further to 29.3 ± 1.5 at day 360 (n = 5), for an overall BMI reduction of 15.1 ± 1.0 kg/m^2^ (p < 0.001). Figure 5b and c depict concomitant progressive increases in TWL and EWL. At day 180, mean TWL and EWL were 28.1 ± 1.0 and 66.2 ± 3.4, respectively (n = 24); at day 360, 34.0 ± 1.4 and 80.2 ± 6.6 (n = 5). All 24 patients (100.0%) surpassed the efficacy criterion of > 5.0% TWL at day 180, and achieved significantly more success with TWL ranging from 20. to 37.6%.

**Table 2:** Evolution of weight and clinical parameters after side-to-side magnetic duodeno-ileostomy with sleeve gastrectomy

	Baseline	6-month follow-up (n = 24)			12-month follow-up (n = 5)		
	Mean ± SEM	Mean ± SEM	Mean change ± SEM (95%CI)	P-value	Mean ± SEM	Mean change ± SEM (95%CI)	P-value
Weight							
Absolute wt, kg	121.9 ± 3.3	87.8 ± 2.8	34.2 ± 1.6 (30.9, 37.4)	< 0.001	77.6 ± 4.7	40.0 ± 3.1 (31.4, 48.6)	< 0.001
BMI, kg/m^2^	44.4 ± 0.8	32.0 ± 0.8	12.4 ± 0.5 (11.5, 13.3)	< 0.001	29.3 ± 1.5	15.1 ± 1.0 (12.2, 18.0)	< 0.001
TWL, %	–	28.1 ± 1.0	–	–	34.0 ± 1.4	–	–
EWL, %	–	66.2 ± 3.4	–	–	80.2 ± 6.6	–	–
Clinical							
HbA1_C_, %*	6.2 ± 0.3	5.1 ± 0.2	1.1 ± 0.4 (0.2, 1.9)	< 0.05	4.8 ± 0.2	2.0 ± 1.1 –^††^	0.173
Glucose, mg/dL^†^	111.3 ± 6.1	86.5 ± 3.5	24.8 ± 6.6 (11.0, 38.6)	< 0.001	87.3 ± 6.3	53.8 ± 6.3 –^††^	0.113

*BMI*: Body mass index; *TWL*: Total weight loss; *EWL*: Excess weight loss; *HbA1*_*C*_: Glycosylated hemoglobin

^*^HbA1_C_ baseline n = 20; 6-month n = 19; 12-month n = 4

^†^Glucose baseline n = 21; 6-month n = 21; 12-month n = 4

^††^Not applicable due to small sample size

#### Metabolic indicators

**Fig. 6:**
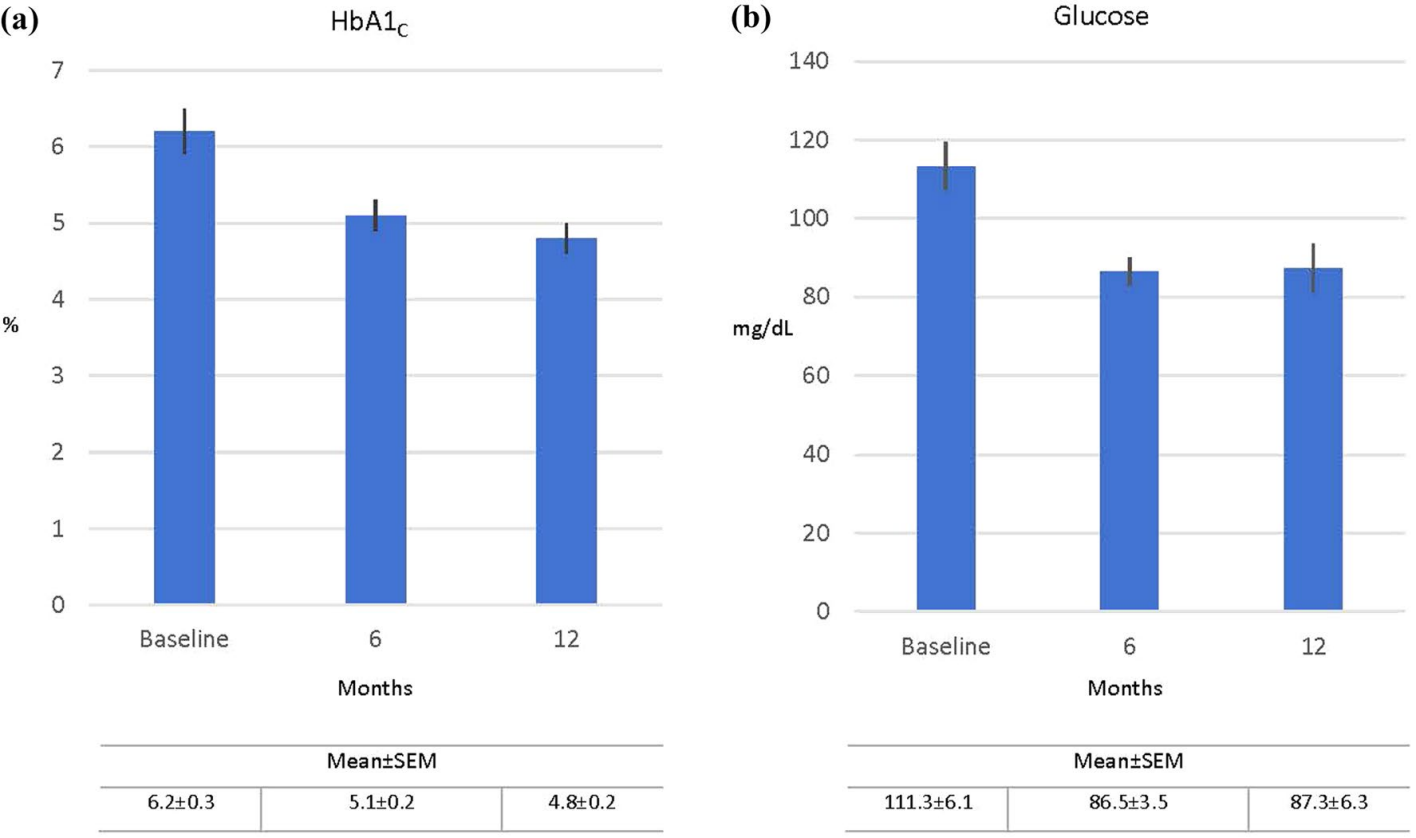
Mean changes following side-to-side Magnet System duodeno-ileostomy with sleeve gastrectomy in **(a)** HbA1_C_ (%): reduction at 6 months, 1.1 ± 0.4% (n = 19) and at 12 months, 2.0 ± 1.1% (n = 4); and in **(b)** blood glucose (mg/dL), reduction at 6 months, 24.8 ± 6.6% (n = 21) and at 12 months, 53.8 ± 6.3% (n = 4)

Figure 6a and b depict changes in HbA1_C_ (n = 19) and glucose levels at postoperative day 180 (n = 21) and day 360 (n = 4), respectively; blood draw compliance was not complete for every patient. Group mean HbA1_C_ level was reduced from 6.2 ± 0.3% at baseline to 5.1 ± 0.2% at day 180, representing a significant overall mean change of 1.1 ± 0.4% (p < 0.05) (Table 2). For the 4 patients who reached the 360-day follow-up timepoint, HbA1_C_ fell to 4.8 ± 0.2, an overall reduction of 2.0 ± 1.1%. Mean glucose level was reduced from 111.3 ± 6.1 mg/dL to 86.5 ± 3.5 mg/dL at day 180, a significant overall mean change of 24.8 ± 6.6 mg/dL (p < 0.001); at day 360 glucose fell to 87.3 ± 6.3 mg/dL, an overall reduction of 53.8 ± 6.3 mg/dL (Table 2).

Seven of 9 patients diagnosed with T2D were under treatment with antidiabetics at enrollment. One hundred percent (7/7) of T2D patients noted less need for medication, with six patients (85.0%) ceasing all medications and one patient reducing medication at day 180. Specifically, four patients stopped medications at enrollment. One patient entered the study taking 3 diabetes medications but stopped 2 immediately (empagliflozin, gliclazide), and stopped the third (metformin) 2 weeks post procedure. Another patient on 2 medications immediately stopped semaglutide, continuing metformin until discontinuing it at 4 months post procedure. The last of the seven patients was on 3 medications and stopped 2 immediately (liraglutide and dapagliflozin), continuing metformin through follow-up. Of the two patients entering with no prescribed T2D medication, one remained off medication, and the other started metformin 10 days post procedure, continuing through follow-up.

### Safety

There were no anastomotic bleeds, leaks, obstruction, infection and no deaths throughout the study. In 21 patients, a total of 57 adverse events was reported, none related to the device. AEs comprised 25 CDC grade I (43.8%), 22 grade II (38.6%), and 10 grade III (17.5%). Included among minor and moderate CDC grade I and II AEs were cases of Covid-19, nausea, diarrhea, vomiting, abdominal pain, an esophageal mucosal tear from blind insertion of the overtube, wound pain, vitamin/mineral deficiency, dehydration, pulmonary and urinary infection, and reflux.

Three SAEs (5.3%, 3/57) were determined to be related to the study procedure, none to the MS. One event was a urinary tract infection with mild fever on postoperative day one; the patient was treated with antibiotics and recovered without sequelae. The second event was a small jejunal obstruction due to an internal hernia in the mesentery, despite the per-protocol closure of the mesenteric defect during the procedure. Laparoscopic repair was performed, and the patient discharged the next day without sequelae. The third event was a pelvic fluid collection that continued for 2 months. After transvaginal drainage, the patient recovered and was in good condition. The 8 SAEs of CDC grade II or III are detailed in Table 3. There were no grade IV or V AEs.

**Table 3:** Clavien-Dindo Classification grade II and III severe adverse events (SAEs) following side-to-side magnetic duodeno-ileostomy with sleeve gastrectomy through 1 year

Event	CDC Grade	Magnet related	Description
Urinary tract infection	II Mild	No	Patient presented with fever on D1, prolonging hospitalization. Urinary analysis confirmed infection; treated with 1 dose Fosfomycin; resolved with no sequelae
Dehydration	II Moderate	No	Patient hospitalized for dehydration and hypokalemia outside country 67 days (holiday); hospitalized again for nausea, vomiting, abdominal pain with suspicion of gastritis; hypokalemia was supplemented, event resolved
Post-SG ano-rexia + diarrhea, nausea, vomiting	II Moderate	No	Patient presented with diarrhea 54 days post procedure: stools watery, not bloody, ≥ 7 episodes/. 2 days later, vomiting began with dehydration, anorexia, dizziness. Abdominal CT showed no features or perforation. Hospitalized 24 h for monitoring with rehydration, IV antiemetics. Symptoms resolved without sequelae
JI obstruction on flange	III Mild	No	At procedure, mesenteric defect closed per protocol. Patient presented 115 days later with occlusion of small intestine by internal hernia in mesentery. Laparoscopic repair performed; discharged 2nd day without sequelae
Major pneumoperi-toneum on gastric fistula	III Severe	No	D2, fever (38.5 °C) developed, antibiotics started. Abdominal CT revealed major pneumoperitoneum. No objectified leakage found on exploratory laparoscopy. Patient developed sepsis; was started on amukin. Thoraco-abdominal CT (injection + barium) showed no leakage or infiltration, but with bi-basal pneumonia; treated with antibiotics. CT scan with gastrografin revealed fistula on left edge of SG + localized abscess. Stents placed and removed. Esophageal prostheses placed in lower esophagus and at EGJ. Naso-jejunal tube placed, and replaced with central line + parenteral nutrition; all later removed. Persistent para-esophageal fistula with leak. Esophageal stents removed; two stents placed in fistula. Abdominal CT with no major findings; patient discharged. SAE determined to be post-SG gastric fistula with favorable evolution after 3 months of multiple hospitalizations and treatments
Pelvic collection	III Severe	No	On D2, patient developed fever with inflammatory syndrome, tachycardia, and desaturation (7–18–22). CT showed free liquid in pelvis; antibiotics started. Patient feeling slightly better, but with persistent inflammatory syndrome; 2nd CT showed pelvic collection which was drained in surgery transvaginally under general anesthesia. Procedure complicated by bleeding 2 days after; gynecology team put stiches at vaginal incision. After 2nd fever spike, antibiotics changed. After good evolution, antibiotics stopped, and patient discharged (8–11–22) in good general condition. Patient presented to Emergency Room 10–24–22 with fever, reporting purulent vaginal bleeding that stopped 48 h before, coinciding with start of fever. She was admitted to hospital and seen by the gynecology department. CT showed pelvic collection, which was drained transvaginally. She was discharged 10–1–22 in good general condition. The source of pelvic collection was not ascertained
Cholecysto-lithiasis + choledocho-lithiasis	III Severe	No	Patient presented July 2022 with abdominal pain in upper right quadrant approx. 2 months after procedure. Investigations showed choledocholithiasis with gallstones in gallbladder. Patient underwent ERCP + sphincterotomy in July and was scheduled for cholecystectomy in October. In August, another episode of choledocholithiasis; 2nd ERCP performed in September with cholecystectomy the following day
Abdominal pain + nausea and vomiting	III Severe	No	Post procedure 4 mo., patient presented with complaints of abdominal pain on right side predominantly increasing for 2 days. Unable to eat/drink for 2 days, with this difficulty since surgery. Hospitalized for treatment; gastroscopy performed. Pain determined unrelated to study device or procedure

*MS*: Magnet System; *DI*: Duodeno-ileostomy; *SG*: Sleeve gastrectomy; *JI*: Jejunoileal; *D*: Day; *EGJ*: Esophagogastric junction; *ERCP*: Endoscopic retrograde cholangiopancreatography

**Clavien-Dindo Classification of surgical complications** [23]: **Grade I:** Deviation from the normal postoperative course without the need for pharmacological treatment or surgical, endoscopic, and radiological interventions. Antiemetics, antipyretics, analgesics, diuretics and electrolytes, and physiotherapy allowed. **Grade II:** Requiring pharmacological treatment with drugs other than such allowed for grade I complications. Blood transfusions and total parenteral nutrition included. **Grade III:** Requiring surgical, endoscopic, or radiological intervention. **Grade IV:** Life-threatening complication (including certain central nervous system complications) requiring Intermediate Care/Intensive Care Unit-management. **Grade V:** Death of patient

## Discussion

The side-to-side Magnet Anastomosis System DI procedure was a feasible technique for surgeons and multidisciplinary MBS teams at three sites. The linear MS device was readily placed, well tolerated in the bowel, and successful in forming patent, sizeable, enduring duodeno-ileal bipartitions without anastomotic leakage through 1-year follow-up. In addition, duodeno-ileostomies were incisionless and suture/staple free. The fused magnets were expelled naturally without migration, erosion, or pair separation. At 6- and 12-month follow-up, substantial weight loss and BMI reduction were observed with 86.0% T2D resolution/100.0% improvement (all T2D medications eliminated [n = 6]) or reduced [n = 1]). Over 1 year, there were 57 total AEs; although none were device related, 8 were determined to be SAEs of CDC grade II or III.

The clinical feasibility of using magnets to effect patent anastomoses in a side-to-side DI can only be directly compared at this time to a feasibility study in 8 patients (4/8 females, median BMI 38.8) of “self-forming magnets (SFM)” by Schlottmann et al. In their series with 1-year follow-up, two octagonally shaped magnets were aligned in the duodenum and ileum, and expelled naturally without device-related AEs. Patent anastomoses were achieved with the proximal SFM magnet delivered by upper endoscopy and the distal magnet by laparoscopy through a 5-mm ileotomy closed with absorbable suture [25]. The SFM enterotomy introduces potential for a leak at the DI site; whereas the MS is positioned without incision or sutures/staples. The devices cannot be compared in terms of efficacy as MS DI was combined with SG in the current study.

The end-to-side SADI-S is the closest comparator to the side-to-side MS DI + SG. Operative time, weight loss, and metabolic outcomes in the magnetic DI + SG study were effective and roughly equivalent to those of SADI-S in three recent observational studies and a meta-analysis. In a prospective short-term comparative study of SADI-S (n = 42) and DS (n = 20) by Andalib et al. (2021), operative time was briefer with primary SADI-S (median 211 min) than for DS (250 min), p = 0.05; whereas, in the current study of side-to-side MS DI + SG, operative time was less (median of 130 min) than for both SADI-S and DS in the Andalib report [26]. Although no randomized controlled trial (RCT) evidence for SADI-S exists currently, a systematic review and meta-analysis by Verhoeff et al. (2022) of 16 studies, 1704 (51.3%) of primary SADI-S compared with other hypoabsorptive procedures, the weighted mean operative time of 103.6 min [27] was less than that of the other hypoabsorptive operations compared in their study and that of the current MS DI + SG operation.

The median EWL of primary SADI-S in Andalib et al. at 6- and 12-month follow-up was, respectively, 72.6% and 86.8% with BMI of 32.9 and 29.4 [26]. In comparing SADI-S vs DS at 2-year follow-up, Verhoeff et al. found their TWL and BMI similar (respectively, 37.3% and 29.8 vs 37.6% and 31.1) [27]. In the current study of magnetic DI + SG, patients with 1-year follow-up (n = 5) had a somewhat lower TWL of 34.0% (EWL 80.2%), and virtually the same BMI of 29.3 found in the Verhoeff et al. meta-analysis. Sanchez‑Pernaute et al., innovators of the SADI-S, recently reported (2022) the largest consecutive series (n = 164) of SADI-S (5-year follow-up [n = 139, 84.7%] with some patients followed through 10 years), respective 1-year TWL, EWL, and BMI were 42.0%, 95.5%, and 26.5 [28]. Whereas, at 1 year, magnetic DI + SG TWL and EWL were markedly lower than those of Sanchez-Pernaute et al., they were still highly significant in our small cohort.

T2D resolution in the current study was 86.0% (n = 6/7 off all medications) at 6 months and 100.0% at 1 year (n = 3/3 off all medications). In the comparative study of SADI-S and DS by Andalib et al., the T2D resolution rate was a median of 91.0% (n = 21/23 at 10 months) and 50.0% after DS (n = 3/6 at 14 months) [26]. The Verhoeff et al. meta-analysis weighted mean remission rate for T2D of 87.4% following SADI-S (measured at longest follow-up, 28.8 months) [27] was similar to that of the current study at 1-year follow-up notwithstanding its small sample size. In a prospective multicenter study of primary SADI-S by Cottam et al. (2020) at 1-year follow-up, in SADI-S patients, T2D in 54/57 of patients (96.0%) was either resolved or improved with continued medications [29]. In relation to recent studies of SADI-S (and SADI-S vs DS), side-to-side MS DI + SG appeared to achieve a comparable rate of T2D resolution.

Side-to-side MS DI + SG had a 0.0% anastomotic leak rate through 1-year follow-up. In the meta-analysis by Verhoeff et al., 10 duodeno-ileal anastomosis leaks (6/16 studies) were reported [27]. Andalib et al. described 1 leak two days after SADI-S at the duodeno-ileal site requiring reoperative repair and drainage [24]; and in Sanchez-Pernaute et al., 2 leaks at the duodeno-ileostomy, one requiring reoperation [28]. While the current study had technical success in magnetic anastomosis formation without leak and reoperations, the SAEs sustained represent an area of challenge requiring future improvement.

Creating and protecting a patent, continuously functioning, non-inflammatory anastomosis is critical to MBS. Compromised anastomotic healing may cause a leak, resulting in potential sepsis or death [30, 31]. The human burden and financial costs are significantly elevated for MBS patients who suffer anastomotic leaks [32]. Use of sutures/staples for anastomosis formation may negatively impact the blood supply in GI tissues. If a stapler is too small for the site, anastomotic stenosis can result. An intestinal fold inadvertently embedded in a sutured/stapled anastomosis can form granulation tissue postoperatively. In addition, GI staples will affect subsequent computed tomography and magnetic resonance imaging examination and treatment [18].

MIS approaches that employ delayed anastomosis technology (DAT), a concept developed by Gagner (2021), aim to alleviate acute and chronic inflammatory responses associated with instant intraoperative anastomosis creation using foreign materials [33]. The MS is a device and approach that embodies DAT. The unhurried DAT paradigm is nonetheless efficient, as gradual healing optimizes conditions for collagen deposition. Patients may require less anesthesia, operative time, and hospital stay, and need fewer reinterventions due to anastomotic complications.

Technical difficulties associated with stapled/sutured anastomoses may be mitigated by magnets combined with single-anastomosis procedures. Feasibility and therapeutic value of employing MCA in other digestive tract indications are being explored and have shown early effectiveness, such as in complex pediatric ileal resection where temporary magnets have been used to create inter-intestinal anastomoses over 5–7 days to treat intestinal invagination. In addition to magnets’ mechanical induction of an anastomosis, evidence suggests that their magnetic field provides physiotherapeutic stimulation of the microcirculation contributing to anastomotic tissue regeneration. [34, 35]. In the context of complex esophageal atresia, endoscopic MCA has been used to achieve delayed primary esophageal repair [36]. Also, in small cohorts, GI fistula, cancer, obstruction, ulcer, and patients with complex abdominal histories have been treated feasibly and safely with MCA [37, 38].

### Limitations

A limitation of this study was its lack of a comparison group. The cohort was multi-national but relatively small, and the design observational; therefore, generalization of efficacy results beyond establishing preliminary trends was not appropriate. The current study protocol was not designed to compare the change in nutritional variables experienced following side-to-side MS DI + SG; doing so in future research would elucidate the nutritional efficacy of this procedure compared with that of SADI-S.

## Conclusions

MCA can offer a further advancement of minimally invasive MBS. In a small multi-center study, the incisionless, sutureless Magnet System was a feasible and safe method of anastomosis formation in side-to-side duodeno-ileal diversion. Side-to-side Magnet System DI + SG proved effective in reducing obesity and T2D at interim timepoints through 1-year follow-up. Larger cohorts and RCT designs are needed to assess MCA safety and effectiveness.
